# Knowledge, Attitude and Practices Regarding Tick‐Borne Diseases Among Healthcare Workers in Two Hospitals in Kilimanjaro, Tanzania: A Cross‐Sectional Study

**DOI:** 10.1155/japr/3390133

**Published:** 2026-07-03

**Authors:** Elichilia R. Shao, Jaffu Chilongola, Rebecca F. Bodenham, Sarah Cleaveland, Tito J. Kibona, Eliaichi A. Mlay, Laura J. Shirima, Innocent B. Mboya, Daniel Mujuni, Rehema A. Mavura, Zacharia L. Laizer, Edna-joy Munisi, Jeremia J. Pyuza, Felix Lankester, Ray Kayaga, Johana Teigen, Blandina T. Mmbaga, Melinda K. Rostal

**Affiliations:** ^1^ Internal Medicine Department, Kilimanjaro Christian Medical Centre, Moshi, Tanzania, kcmc.ac.tz; ^2^ Kilimanjaro Clinical Research Institute, Moshi, Tanzania, kcri.ac.tz; ^3^ Internal Medicine Department, School of Medicine, KCMC University, Moshi, Tanzania, kcmuco.ac.tz; ^4^ Department of Medical Biochemistry and Molecular Biology, School of Medicine, KCMC University, Moshi, Tanzania, kcmuco.ac.tz; ^5^ School of Biodiversity, One Health and Veterinary Medicine, University of Glasgow, Glasgow, UK, gla.ac.uk; ^6^ Global Health Tanzania, Arusha, Tanzania; ^7^ Department of Life Science and Bioengineering, Nelson Mandela African Institution for Science and Technology, Arusha, Tanzania; ^8^ Department of Prosthetics and Orthotics, School of Rehabilitation Medicine, KCMC University, Moshi, Tanzania, kcmuco.ac.tz; ^9^ Department of Epidemiology and Biostatistics, School of Public Health, KCMC University, Moshi, Tanzania, kcmuco.ac.tz; ^10^ Department of Research and Programs, Africa Academy for Public Health, Dar es Salaam, Tanzania; ^11^ Community Health Department, Kilimanjaro Christian Medical Centre, Moshi, Tanzania, kcmc.ac.tz; ^12^ Department of Microbiology and Immunology, School of Diagnostic Medicine, Muhimbili University of Health and Allied Sciences, Dar es Salaam, Tanzania, muchs.ac.tz; ^13^ Internal Medicine Department, Mawenzi Referral and Regional Hospital, Moshi, Tanzania; ^14^ Pathology Department, School of Medicine, KCMC University, Moshi, Tanzania, kcmuco.ac.tz; ^15^ Paul G. Allen School for Global Health, Washington State University, Pullman, USA, wsu.edu; ^16^ Tanzania Veterinary Laboratory Agency, Arusha, Tanzania; ^17^ One Health Research Consulting, Glen Rock, New Jersey, USA; ^18^ Pediatric and Child Health Department, School of Medicine, KCMC University, Moshi, Tanzania, kcmuco.ac.tz; ^19^ Department of Production Animal Studies, Faculty of Veterinary Science, University of Pretoria Onderstepoort, Pretoria, South Africa

## Abstract

Tick‐borne diseases (TBDs) are significant causes of febrile illnesses in humans. Healthcare workers′ (HCWs) knowledge, attitudes, and practices (KAP) regarding ticks and TBDs may influence prognosis. This study is aimed at assessing the KAP and associated factors among HCWs regarding TBD to provide evidence to improve preventive strategies, diagnosis, and enhance patient outcomes. The electronic questionnaires were used to collect data on respondents′ demographics and KAP on TBD. Of 401 HCWs, the median age was 29 (interquartile range (IR): 26–39). More than half of all HCWs (58.4%) were aged ≤ 30 years, and 51.1% were male. Only 12.2% received training outside Tanzania, 45.4% were medical doctors. Among all HCWs, 47.1% had TBD knowledge above the median. Regarding attitude of HCWs toward TBD, 208 (97.7%) out of 214 who had some awareness of TBD recognized the knowledge gap and a need for further training on TBD. After adjusting for other factors, being male (adjusted odds ratio (AOR) = 1.57, 95% CI: 1.01–2.46), having a postgraduate education (AOR = 3.05, 95% CI: 1.45–6.39), and having received TBD training (AOR = 2.08, 95% CI: 1.28–3.40), remained significantly associated with TBD knowledge above median. Similarly, after adjusting for other factors, being male (AOR = 0.57, 95*%*CI = 0.37–0.89), having postgraduate education (including physicians; AOR = 0.28, 95*%*CI = 0.13–0.61), and having awareness on TBD were significantly associated with practice above median toward TBD prevention. The knowledge gap on TBD among HCWs was substantial as more than half of the HCWs had knowledge score below median about TBD. On attitude, almost all HCWs who had some awareness of TBD were in need of training on TBD. More than half of HCWs practiced TBD preventative actions below median. Targeted training to improve and expand HCWs′ knowledge, attitudes, and practices regarding TBD are expected to enhance early detection, improve patient outcomes, reduce transmission rates, and strengthen public health response efforts.

## 1. Introduction

Ticks are arthropod vectors that can transmit human and animal pathogens [1]. Tick‐borne diseases (TBDs) are a global concern and many are emerging and re‐emerging zoonotic diseases [2]. Limited awareness of healthcare providers, inappropriate and inaccessible diagnostic tools, and the absence of a surveillance system for reporting TBDs may lead to underestimating prevalence in humans [3–6]. TBDs may present with nonspecific clinical signs and symptoms; therefore, proper history‐taking and the selection of appropriate diagnostic tests are crucial for accurate diagnosis and effective treatment, which in turn may result in fewer complications and lower mortality [7]. Some of the TBDs, such as Rocky Mountain spotted fever (RMSF), are fatal in up to 30% of cases, whereas the fatality rate of Crimean‐Congo hemorrhagic fever virus (CCHFV) may reach 80% during an outbreak [8]. Studies in Dodoma, Arusha, and Kilimanjaro, Tanzania, have detected tick‐borne pathogens (TBPs), such as *Borrelia duttonii*, spotted fever group *Rickettsia* and *Coxiella burnetii* [9–12]. The most common signs and symptoms are headache, joint and back pain, fever, and vomiting [9, 10, 13]. These nonspecific clinical presentations pose a significant challenge to clinicians, especially in areas with limited laboratory diagnostic capacity for febrile illnesses, leading to poor outcomes and prognosis [14, 15]. To reduce these unintended outcomes, HCWs should have adequate knowledge and skills to maintain a high index of suspicion and be aware of the possible tick exposure risks to patients. Correct case management should also accompany an early and accurate diagnosis [16].

Few studies have examined knowledge, attitudes, and practices (KAP) regarding ticks and TBDs among HCWs, with more studies focused on veterinarians, hikers, miners, farmers, environmental experts, and general population [7, 17–19]. A recent study in Rwanda among HCWs and the general population showed a significant knowledge gap and negative attitudes where even after tick bites people do not seek health services until when they are sick [20]. Other studies in Canada on TBD KAP among residents with a high risk of TBDs have shown a low level of adoption of preventive behaviors, indicating a need to improve knowledge and promote behavior changes [21]. HCWs often see patients with nonspecific symptoms, some of whom may have TBD‐related infections. Their knowledge above median will improve the quality of patient history‐taking, which helps narrow down the list of possible diagnoses. When combined with a favorable attitude and practice with local facilities, this knowledge may help establishing differential diagnosis for conditions like CCHFV, RMSF, and anaplasmosis. These efforts could lead to better disease outbreak control and prevent secondary transmission [22, 23]. Other factors such as changes in land use, habitat alterations, altered host dynamics, and human behaviors due to climate change, have been linked to shifts in the incidence of TBD [24]. Climate change affects tick proliferation rates, changes in transmission seasons, the migration of humans, birds, and wildlife animals, and change in habitable range, all of which can lead to TBD becoming prevalent in nonendemic regions [21–23].

TBD among livestock—including East Coast fever, anaplasmosis, and babesiosis—has been studied in Tanzania, with limited studies from humans even in the presence febrile illnesses of unknown causes [14, 25], which calls for a deeper understanding of the KAP regarding ticks and TBD among HCWs [26]. Evaluating HCW′s knowledge of TBD is crucial, as it helps identify gaps in understanding that may influence the timely and accurate diagnosis and treatment of these conditions. This assessment is essential because it ensures that HCWs possess the knowledge necessary to provide prompt interventions, minimize complications, and ultimately improve patient outcomes [4, 19, 22]. Limited TBD knowledge has been associated with inadequate training among physicians as well as general population to prevent spillover effects [19]. Despite significant advancements in surveillance systems, improved diagnostic modalities, treatment, and preventive measures, these efforts are not well coordinated, especially in high‐endemic areas [22, 24]. Evidence suggesting a lack of awareness regarding ticks and TBD among HCWs in Tanzania [10, 11, 27] and the presence of human TBDs in the northern zone of the country [10, 13], hence assessing the knowledge of HCWs regarding ticks and TBDs is crucial toward early and correct diagnosis and treatment of these conditions and will lead to better patient outcomes.

## 2. Materials and Methods

### 2.1. Study design, population, and setting

We conducted a cross‐sectional study between November 20 and December 20, 2023, among HCWs from Kilimanjaro Christian Medical Centre (KCMC), a 630‐bed zonal referral hospital, and from Mawenzi Regional Referral Hospital (MRRH), a 300‐bed regional referral hospital, both located in Moshi Municipality, Kilimanjaro, Tanzania. KCMC and MRRH were purposefully selected because they are the two referral hospitals involved in teaching students and consist of the most experienced HCWs in the Kilimanjaro region. The study included clinicians/medical doctors (bachelor′s degree or higher), registered nurses (diploma education level or higher), and laboratory scientists (diploma education level or higher). Postgraduates were considered as anyone with a master′s level degree or higher. All HCWs who have practiced for more than 6 months in the selected facilities and provided informed consent were eligible for inclusion. Moshi municipality, with a population of approximately 180,000 people, is the administrative capital of the Kilimanjaro region, with a total population of around 1.6 million. Situated approximately 890 meters above sea level, Moshi has a tropical climate, with rainy seasons occurring from October to December and from March to May [28]. In Tanzania, one out of three people keep livestock and the majority of livestock is managed by small holders [29, 30].

### 2.2. Sample Size and Sampling

A minimum required sample size for this study was estimated by using a single proportion formula given as: *n* = [(*z* *a*/2)2∗*p* (1 − *p*)]/*e*2; whereby, n is the required sample size, z_a/2_ is the standard normal value under 95% confidence level (i.e., 1.96), p is the estimated proportion, that is, 33.3% of HCWs with adequate TBD knowledge (based on a Kenyan study [31], and e is the margin of sampling error (5%). Assuming a 20% nonresponse proportion, the minimum sample size constituted 400 HCWs. The ratio of eligible HCW staff at MRRH (750 workers) to KCMC (1250 workers) was 3:5. To reflect this difference in population size in the study we planned to enroll 60% of the participants from KCMC (240) and 40% from MRRH (160). We used a systematic random sampling to select HCWs from each department′s list of staff (every other employee on each list). All consenting HCWs were enrolled in the study until the required sample size was achieved.

### 2.3. Data Collection

Trained research assistants collected data using a digitized structured questionnaire. The tool was designed and administered in English language depending on the HCWs′ preference, using Open Data Kit software. The questionnaire was piloted among HCWs at Mount Meru Regional Hospital in Arusha, Tanzania, which is a regional level hospital similar to MRRH. The questionnaire data collected included HCWs′ sociodemographic characteristics such as age, gender, education, work experience, and department, and KAP questions on ticks and TBD including types, causes, modes of transmission, signs and symptoms, diagnosis, treatment, prevention, and control. Participants were enrolled during both day and night shifts throughout the study period. All interviews were conducted in a private and secure room within each hospital after HCWs provided informed consent. To ensure confidentiality participant identification numbers were used and names were not recorded. Data entry was double‐checked for any errors by two members of our research team before submission to the server.

### 2.4. KAP Scoring

The outcome variables in this study were knowledge, attitude and practices on TBD. TBD knowledge was measured by asking HCWs questions on: (I) Whether they knew about TBD; (II) Naming at least one TBD they knew; and (III) TBD signs and symptoms, transmission, diagnosis, and prevention measures. Participants were also asked about whether they were taught/trained about TBD, and if so, the courses attended, and institutions offering such courses. For every question asked about transmission, symptoms, diagnosis, and prevention of TBD, the correct response was assigned 1 and for an incorrect response 0. A knowledge score was calculated by summing the participants score across signs and symptoms, transmission, diagnosis, and preventive measures with a maximum score of 36. The median value of 16.9 was used to categorize the overall knowledge score into binary outcome variables (inadequate knowledge < 17 and adequate knowledge ≥ 17). This scoring and cut off point is consistent with other work on Malaria prevention knowledge, attitudes and practices among adolescents living in an area of persistent transmission in Senegal [32].

The attitude of *HCWs toward* TBD was evaluated using the following four questions: (I) Would you like to receive training on ticks and/or TBDs?; (II) Do you think TBDs are a concern in your region of origin?; (III) Is it important to provide patient education about ticks and TBD?; and (IV) Is there any need for public health outreach services on TBDs in your region of origin? The respondents answered *Yes* or *No* to each question.

TBD practices were evaluated using the following six questions: (I) Do you look for ticks on yourself after visiting a place known to have ticks/spend time with (domestic OR wild animals?); (II) When you attend febrile patients, do you ask them about their history of tick exposure?; (III) In your daily practices, do you consider TBD among your differential diagnoses?; (IV) Do you provide information about TBD to your patients?; (V) Do you protect your pets/animals from ticks?; and (VI) Does your institution provide educational materials to the public on TBD? The responses were recorded using the following scoring: 3 (*Every time*), 2 (*Sometimes*), 1 (*Unsure*), and 0 (*Never*), summing up to a maximum score of 18. To calculate the overall practice score, the questions were summed up, thereafter the median value of the calculated practice score was used to categorize the practice score into a binary outcome variable: practice above median (> 8) and 0 for respondents who had a practice below median ≤ 8.

### 2.5. Statistical Analyses

For descriptive statistics, absolute frequencies and percentages were used to summarize categorical variables, whereas mean/median with its standard deviations (SD)/interquartile range were used to summarize continuous variables. Descriptive statistics were used to summarize responses to attitude questions. The chi‐squared test was used to compare the proportion of TBD knowledge and practice by participant characteristics. Logistic regression was performed to identify factors associated with knowledge and practice regarding TBD, estimating odds ratios (OR) with their 95% confidence intervals (CI). All variables with a *p* value of < 0.1 in the bivariate analysis and those with clinical importance were considered for multivariable logistic regression. In multivariable logistic regression analysis for factors associated with TBD knowledge, the independent variables that were analyzed included age (years), sex, education level, professional, years since completing the current highest educational level, years of working experience, current working on the department, and training on TBD. For factors associated with TBD practice, the independent variables analyzed include age (years), sex, education level, profession, years since completing the current highest educational level, years of working, and awareness of TBD. The variable was considered statistically significant at a threshold of 0.05. The Akaike Information Criteria (AIC) was utilized to assess the model fit, considering models with lower AIC values as the best fit. Multicollinearity of independent variables was evaluated using the variable inflation factor (VIF), where a VIF exceeding 10 indicates multicollinearity, with no major violations observed.

## 3. Results

### 3.1. Baseline Characteristics of the HCWs

We sampled a total of 401 HCWs, *n* = 253; (63.0%, 95% CI: 0.58–0.68) from KCMC and *n* = 148; (37.0%, 95% CI: 0.32–0.42) from MRRH. The median age was 29 (IQR: 26–39), with more than half *n* = 234 (58.4%, 95% CI: 0.53–0.63) aged ≤ 30 years. Half of the respondents were male *n* = 205 (51.1%, 95% CI: 0.46–0.55). Medical doctors or specialists were well represented *n* = 182 (45.4%, 95% CI: 0.41–0.50 and *n* = 273 (68.1%, 95% CI: 0.63–0.72) of respondents worked in nonsurgical departments. The majority of participants had less than 10 years of work experience *n* = 316 (78.8%, 95% CI: 0.75–0.83), and *n* = 214 (53.4%, 95% CI: 0.48–0.57) stated they were aware of TBD. A further *n* = 112 (27.9%, 95% CI: 0.24–0.33) had received some training on TBD. Only *n* = 49 participants (12.2%, 95% CI: 0.09–0.16) had received training outside of Tanzania (Table 1).

**Table 1:** Background characteristics of HCWs at KCMC and MRRH in Moshi Municipality, Kilimanjaro Tanzania (*N* = 401).

Variable	Frequency	Percentage (95% CI)
Age (years)		
Median (IQR)	29 (26–39)	
≤ 30	234	58.4 (0.53–0.63)
31–40	78	19.4 (0.16–0.24)
> 40	89	22.2 (0.18–0.26)
Gender		
Female	196	48.9 (0.44–0.54)
Male	205	51.1 (0.46–0.56)
Education level		
Undergraduate	333	83.0 (0.79–0.86)
Postgraduate	68	17.0 (0.14–0.21)
Years since completed education		
< 5	227	56.6 (0.52–0.61)
5–10	90	22.4 (0.19–0.27)
> 10	84	21.0 (0.17–0.25)
Country completed education		
Tanzania	352	87.8 0.84–0.91)
Outside Tanzania	49	12.2 (0.09–0.16)
Profession		
Medical doctor/specialists	182	45.4 (0.41–0.50)
Nurses	163	40.6 (0.36–0.46)
Laboratory scientist	56	14.0 (0.11–0.18)
Health facility		
KCMC	252	63.0 (0.58–0.68)
MRRH	148	37.0 (0.32–0.42)
Current working department		
Surgical	128	31.9 (0.28–0.37)
Nonsurgical	273	68.1 (0.63–0.72)
Working experience (years)		
≤ 10	316	78.8 (0.75–0.83)
> 10	85	21.2 (0.17–0.25)
Awareness on TBD		
Yes	214	53.4 (0.48–0.58)
No	187	46.6 (0.42–0.52)
Trained/attended a course on TBDs		
No	289	72.1 (0.67–0.76)
Yes	112	27.9 (0.24–0.33)
TBD course attended (*N* = 111)		
Formal training	76	68.5 (0.59–0.76)
Short course	21	18.9 (0.13–0.27)
Both	14	12.6 (0.08–0.20)
Organization‐offered TBD (*N* = 110)		
Government	39	35.4 (0.27–0.45)
Nongovernment	71	64.6 (0.55–0.73)

### 3.2. Knowledge of TBDs among HCWs

Of the total 401 HCWs analyzed, less than half *n* = 189 (47.1%, 95% CI: 0.42–0.52) had knowledge above mean on TBD. Among 252 HCWs from KCMC, 46.4% (*n* = 117) had TBDs knowledge above median. Among 89 HCWs aged ≥ 40 years, 56.2% (*n* = 50) had TBDs knowledge above median; among 205 HCWs who were males, 52.7% (*n* = 108) had TBDs knowledge above median. Among 182 doctors, 57.1% (*n* = 104) had TBD knowledge above median, and among 273 HCWs in the nonsurgical, 50.6% (*n* = 138) had TBD knowledge above median. Moreover, among 85 HCWs with more than 10 years of work experience, 60% (51) had knowledge above median on the TBDs, and among 112 HCWs who were trained on the TBDs, 62.5% (*n* = 70) had knowledge above median on the TBDs. In multivariable analysis the independent variables; sex, education level, and training were significantly associated with higher odds of knowledge above the median on TBD. Male HCWs had 1.57 times higher odds of knowledge above median on TBD compared to females (AOR = 1.57, 95% CI: 1.01–2.46).

Additionally, HCWs with a postgraduate education had three times higher odds of knowledge above median as compared to those with an undergraduate degree (AOR = 3.05, 95% CI: 1.45–6.39). Furthermore, HCWs trained on TBD courses had 2.08 times higher odds of knowledge above median compared to those with none (AOR = 2.08, 95% CI: 1.28–3.40). (Table 2).

**Table 2:** Factors associated with knowledge above median and practice above median on tick‐borne diseases among HCWs at KCMC and MRRH in Moshi municipality, Kilimanjaro, Tanzania (N = 401).

	TBD knowledge above median				TBD practice above median			
Variable	COR (95% CI)	*p*	AOR (95% CI)	*p*	COR (95% CI)	*p*	AOR (95% CI)	*p*
Age (years)	1.03(1.00–1.05)	0.017	1.01 (0.97–1.05)	0.555	0.97 (0.95–0.99)	0.007	0.99 (0.96–1.03)	0.752
Gender								
Female	Ref		Ref		1.00 (Ref)		1.00 (Ref)	
Male	1.58 (1.06–2.35)	0.023	1.57 (1.01–2.46)	**0.047**	0.63 (0.42–0.93)	0.020	0.57 (0.37–0.89)	**0.014**
Education level								
Undergraduate	Ref		Ref		1.00 (Ref)		1.00 (Ref)	
Postgraduate	4.65 (2.55–8.48)	< 0.001	3.05 (1.45–6.39)	**0.003**	0.24 (0.13–0.45)	< 0.001	0.28 (0.13–0.61)	**0.001**
Years since completed education								
≤ 10	1.00 (Ref)		Ref		1.00 (Ref)		1.00 (Ref)	
> 10	1.67 (1.02–2.70)	0.040	0.78(0.37–1.64)	0.512	0.43 (0.26–0.73)	0.001	0.68(0.33–1.42)	0.304
Profession								
Nurses/laboratory scientist	1.00 (Ref)		Ref		1.00 (Ref)		1.00 (Ref)	
Medical doctor/specialists	2.10 (1.41–3.14)	< 0.001	1.58 (0.99–2.52)	0.051	0.62 (0.41–0.92)	0.017	0.78 (0.49–1.25)	0.314
Health facility								
KCMC	1.00 (Ref)				1.00 (Ref)			
MRRH	1.06 (0.71–1.60)	0.765			1.20 (0.80–1.80)	0.386		
Current working department							—	
Surgical	1.00 (Ref)		Ref		1.00 (Ref)			
Nonsurgical	1.54 (1.01–2.36)	0.046	1.57(0.98–2.48)	0.055	0.78 (0.51–1.19)	0.255	0.81(0.51–1.27)	0.360
Working experience (years)								
≤ 10	1.00 (Ref)		Ref		1.00 (Ref)		1.00 (Ref)	
> 10	1.93 (1.19–3.15)	0.008	1.02 (0.41–2.55)	0.969	0.42 (0.25–0.71)	0.001	0.71 (0.28–1.76)	0.458
Awareness on TBD								
No					1.00 (Ref)		1.00 (Ref)	
Yes					1.4 (0.98–2.15)	0.065	2.35(1.51–3.66)	**< 0.001**
Trained/attended a course on TBDs							—	
No	1.00 (Ref)		1.00 (Ref)		1.00 (Ref)			
Yes	2.38 (1.52–3.73)	< 0.001	2.08 (1.28–3.40)	**0.003**	0.89 (0.58–1.39)	0.619		

*Note:* Adjusted analysis results from multiple logistic regression models adjusted for TBDs knowledge, models were adjusted for age (years), sex, education level, professional, years since completing the current highest educational level, years of working experience, current working on the department, and training on TBD. Bold formatting was used to highlight statistically significant associations (*p* < 0.05) in both the crude and adjusted logistic regression analyses.

Abbreviations: AOR, adjusted odd ratio; COR, crude odd ratio; KCMC, Kilimanjaro Christian Medical Center; MRRH, Mawenzi Regional and Referral Hospital; TBDs, tick‐borne diseases.

### 3.3. Attitude of HCWs on TBDs

**Figure 1:**
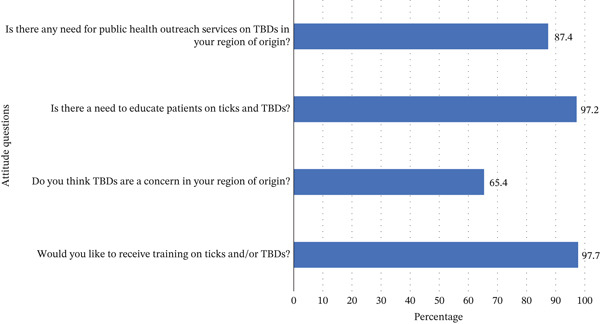
Proportion of HCWs who responded “yes” to each TBD attitudes question. Combined responses from KCMC and MRRH in Moshi municipality, Kilimanjaro, Tanzania (*n* = 214). Note: TBDs, tick‐borne diseases; KCMC, Kilimanjaro Christian Medical Center; MRRH, Mawenzi Regional and Referral Hospital.

Among the 401 HCWs interviewed, *n* = 214 (53.4%) who reported having an awareness of TBD were asked about their attitude toward TBD. Out of them, 187 (87.4%) answered *yes* to the question about the need for outreach services on TBD in the region, 208 (97.2%) responded *yes* about the need to educate patients on ticks and TBDs, 140 (65.4%) thought that TBD were a concern in their region of origin, and 208 (97.7%) wanted to receive tick and TBD training. (Figure 1).

### 3.4. Practices on TBDs Prevention Among HCWs

The overall proportion of HCWs with practice above median toward TBD was 46.6% (95% CI: 0.42–0.51: *n* = 187). In multivariable analysis independent variables sex, education level, and having ever attended a course on TBD had a significant association with practice above median toward TBD. Male HCWs had a lower odds of practice above median toward TBD prevention compared to female HCWs (AOR = 0.57, 95% CI: 0.37–0.89). Compared to HCWs with undergraduate degree, those with postgraduate level of education had 74% lower odds of practice above median toward TBD (AOR = 0.28, 95% CI: 0.13–0.61). HCWs who reported having awareness of TBD had 2.3 times higher odds of practice above normal on TBD compared to those who reported that they were not aware of TBD (AOR = 2.30, 95% CI: 1.47–3.59) (Table 2).

## 4. Discussion

To our knowledge, this is the first study to explore the knowledge, attitude and practices of HCWs regarding TBD in Tanzania, and has revealed significant knowledge and practice gaps that require targeted training. Less than half (47.1%) of HCWs demonstrated knowledge above median on the signs and symptoms, transmission, diagnosis, and prevention of TBD. Factors such as being male, having postgraduate education and previous training on TBD were associated with knowledge above median but surprisingly had lower odds of practices above median on TBD among HCWs. Notably, postgraduate education and prior training on TBD were significantly associated with knowledge above median with 76% of postgraduates showing knowledge above median likely due to increased clinical exposure, involvement in teaching, specialized training such as epidemiology of infectious diseases [33]. The postgraduates are involved in mentoring junior HCWs and medical students which provides them with more opportunities to become more conversant about TBD. Higher education significantly enhances knowledge, emphasizing the importance of advanced training [34]. The focus on TBD‐specific training, research, clinical cases discussion, and journal clubs′ involvement may expose postgraduate students to diverse knowledge, including TBD, resulting in reducing the knowledge gap. In this study, it has been evidenced that attending TBD specific courses had a significant association with knowledge above median, emphasizing the role of continued medical education. This indicates that even if there are no TBD training as a module or within the general curriculum, receiving targeted training can significantly enhance knowledge level. A similar KAP study in China showed that one of the important factors for misdiagnosis of TBD among the undifferentiated febrile patients by HCWs was lack of awareness of TBD [35]. Therefore integrating TBD content into existing in‐service and preservice training program is very crucial to fill the gaps and have competent HCWs.

With less than half of respondents having level of knowledge of TBD above median, the results from this study suggest that there is a TBD knowledge gap in HCWs in the study region. This knowledge gap among HCWs can result in misdiagnosis, leading to poor patient outcomes, especially in resource‐limited settings where laboratory diagnostic capacity is limited. Multiple studies conducted globally on TBD knowledge among HCWs documented varying levels of understanding based on endemicity, professionalism, specialized training, and work experience [34–36]. The knowledge gap among HCWs may arise from a lack of focus on TBD in medical training, limited continuing education opportunities, lack of TBD research, and inadequate exposure to cases in clinical practice due to inability to diagnose TBD [13, 35, 36]. The HCWs′ knowledge on TBD is likely important as it will help clinicians to be able to differentiate TBD from other febrile illness which may have overlapping signs and symptoms [37, 38]. This study found a significant difference on knowledge and practices between genders despite the fact that just over half of all males had adequate knowledge of TBD. Males achieved higher knowledge scores, which aligns with findings from other studies, possibly due to gender inequality in clinical training where there is a male predominance at the postgraduate level although both being male and postgraduate had a lower odds of reporting practice above median. Studies among medical students in Europe, Scandinavia, and Asia on TBD showed that men scored higher in knowledge tests than women [39–41]. Males may be more prevalent in certain specialties with focused training on TBD as compared to females. Another explanation is the difference could be attributed to the cultural context where males are more involved in farming and herding when young before medical school, leading to more exposure to field environment giving them the opportunity for basic knowledge about TBD as compared to females [42]. Data from general population in Scandinavian countries showed no difference between males and females in identifying different species of ticks [43]. The Scandinavian countries have a different culture from Tanzania, with a different environment and different training opportunities for males and females. [33, 36, 44].

The two sites where the study was conducted are different; KCMC is a zonal teaching referral hospital where there are more trained and specialized HCWs as compared to MRRH but surprising there was no significant difference in TBD knowledge level among HCWs at the two hospitals. This might be explained by the lack of targeted training, clinical priorities (focus on other diseases that is HIV, TB, cardiovascular diseases) and self‐directed learning gap (using similar policy and national guidelines which is missing TBD management components) [37, 45]. It might be explained by the absence of on‐the‐job training on TBD among HCWs; hence, there is no significant difference between HCWs who are above or below 40 years. The study in China among HCWs showed different findings that old age was associated with better knowledge on TBD among HCWs [36]. The difference between our study and the study in China might be due to different focus on TBD training, availability of TBD management guidelines, access to TBD training materials, as well as disease endemicity as compared to Tanzania. Furthermore, poor dissemination of the limited existing research data on TBD, and challenges in laboratory diagnosis may hinder knowledge acquisition and result in inadequate knowledge among HCWs [45]. Other contributing factors might be insufficient institutional support for TBD‐related education and a low perceived risk of TBD among HCWs, especially in nonendemic or resource‐constrained settings [45].

Despite gaps in knowledge and practices toward TBD diagnosis and prevention, HCWs′ attitudes toward TBD were generally favorable, with many expressing a willingness to pursue further training on TBD. HCWs need training to understand TBD diagnosis, treatment and prevention especially in areas where undiagnosed febrile patients present with similar signs and symptoms [15]. The majority of HCWs had favorable attitude about providing public health education about ticks and TBD to their clients and also in advocating for outreach programs. Even with the knowledge gap, HCWs believed that ticks and TBDs are a concern in the region where they come from. Although these HCWs were committed to patient care and public health, which fosters their favorable attitude toward learning and addressing TBD, favorable attitudes alone will be insufficient to promote effective practices. Willingness to learn indicates that HCWs recognize the significant gaps in the diagnosis and prevention of TBD and are motivated to enhance their competencies when given the opportunity [42]. HCWs′ favorable attitudes may originate from their daily practices, where they see the gaps in proper diagnosis of undifferentiated infectious diseases as well as its prevention. This favorable attitude provides a foundation upon which targeted training can be implemented to build HCWs capacity and fill their knowledge gap. Favorable attitudes reflect an openness among HCWs to engage with TBD prevention efforts if given the right tools and support.

Approximately 46.6% of the HCWs had scores indicating they had practices above median with regards to TBD prevention and diagnosis, and this was associated with gender, education level, and being aware that TBD exists. Male HCWs, despite potentially having knowledge above median, have practices below the median toward TBD prevention [35, 43]. This paradox suggests that knowledge above median does not necessarily translate into good practical application, possibly due to a lack of hands‐on training or emphasis on clinical prioritization over preventive measures. Females might be good in preventive practices due to differences in risk perception, with female HCWs potentially being more cautious hence more likely to use personal protection measures such as wearing protective clothing or using repellents as compared to males. Low perceived vulnerability of males toward their risks to TBD might lead to practices below median as compared to females. TBD studies in the general population in Scandinavian countries have shown that males aged 18–29 years had knowledge above median and practices above median on TBD [39]. Male HCWs, despite potentially having more knowledge, might not translate it into active preventive measures, possibly due to lower perceived vulnerability or different clinical priorities [42, 43]. HCWs with bachelor education level who see general patients showed preventive practices above median which might be explained by their engagement with general patient care, including febrile cases; hence, being more knowledgeable and sensitive to preventive measures rather than specialized care HCWs [42, 46]. Prior awareness of TBD was associated with practices above median which put emphasis on the role of prior knowledge and its impact on good practices toward prevention and diagnosis of TBD [43, 46]. This reiterates the need for continued medical education and refresher courses to improve clinical care and preventive services on TBD.

This study is the first to assess knowledge, attitude, and practices of TBD among HCWs in Tanzania, revealing significant gaps that require targeted training. Limitations of this study include the reliance on self‐reported data which may introduce response bias, as HCWs may overestimate their knowledge or adherence to best practices. Additionally, the study sample may not fully represent all HCWs, particularly those in urban or remote settings or subspecialties less commonly involved in TBD management. The findings may be generalized to the wider population of HCWs in other referral hospitals in Tanzania.

## 5. Conclusion

This study highlights significant gaps in the KAP of HCWs regarding TBD diagnosis and management in the Kilimanjaro region. Improving the management of nondifferentiated febrile illnesses in Tanzania requires strong clinical awareness of TBD and thorough patient history‐taking, particularly regarding tick exposure. TBD should routinely be included in differential diagnoses, and testing should be pursued whenever possible. When diagnostics are unavailable, collaborating with regional or national health facilities to enhance access is essential. Knowledge alone is insufficient; HCWs need the tools and systems in place to take action. Enhanced training must be coupled with improved diagnostics and clear national guidelines to support the effective diagnosis, prevention, and treatment of TBD.

## Author Contributions

E.R.S., B.T.M., J.C., S.C., F.L., M.K.R., R.F.B., R.K., E.A.M., L.J.S., J.J.P., I.B.M., R.A.M., D.M., Z.L.L., T.J.K., and J.T. designed the study, which included reviewing the pilot work. E.R.S., E.A.M., J.P., R.M., E.M., and T.B. were involved in data collection. E.R.S., B.T.M., J.C., E.A.M., L.J.S., I.B.M., M.K.R., R.F.B., and J.T. participated in data cleaning and analysis. E.R.S. wrote the first draft, and all authors reviewed drafts and provided feedback.

## Funding

This study was supportd by the US Department of Defense, Deference Threat, Grant #HDTRA1‐20‐1‐0018,

## Disclosure

All authors read and approved the final version.

## Ethics Statement

Ethical clearance was granted by the Medical Research Coordinating Committee of the National Institute of Medical Research in the United Republic of Tanzania (NIMR/HD/R.8a/Vol.IX/3596). This research was conducted in accordance with the Declaration of Helsinki. All HCWs provided oral informed consent before the interviews. Confidentiality and identity protection were ensured from data collection stage where identification numbers (IDs) were used instead of names toward analysis and interpretation of the study findings.

## Conflicts of Interest

The authors declare no conflicts of interest.

## Data Availability

Data are available for sharing upon request by the reviewers and/or editor.
